# Swelling Degree of Polyelectrolyte Layers Determined by an Electrochemical Quartz Crystal Microbalance

**DOI:** 10.1021/acs.biomac.4c01205

**Published:** 2025-01-22

**Authors:** Christian Leppin, Agata Pomorska, Maria Morga, Pawel Pomastowski, Piotr Fijałkowski, Aneta Michna, Diethelm Johannsmann

**Affiliations:** † Institute of Physical Chemistry, Clausthal University of Technology, Arnold-Sommerfeld-Str. 4, 38678 Clausthal-Zellerfeld, Germany; ‡ Jerzy Haber Institute of Catalysis and Surface Chemistry, Polish Academy of Sciences, Niezapominajek 8, PL-30239 Krakow, Poland; § Centre for Modern Interdisciplinary Technologies, Nicolaus Copernicus University, Wilenska 4, 87 100 Torun, Poland; ∥ Department of Inorganic and Coordination Chemistry, Nicolaus Copernicus University in Toruń, Gagarina 7, 87-100 Torun, Poland

## Abstract

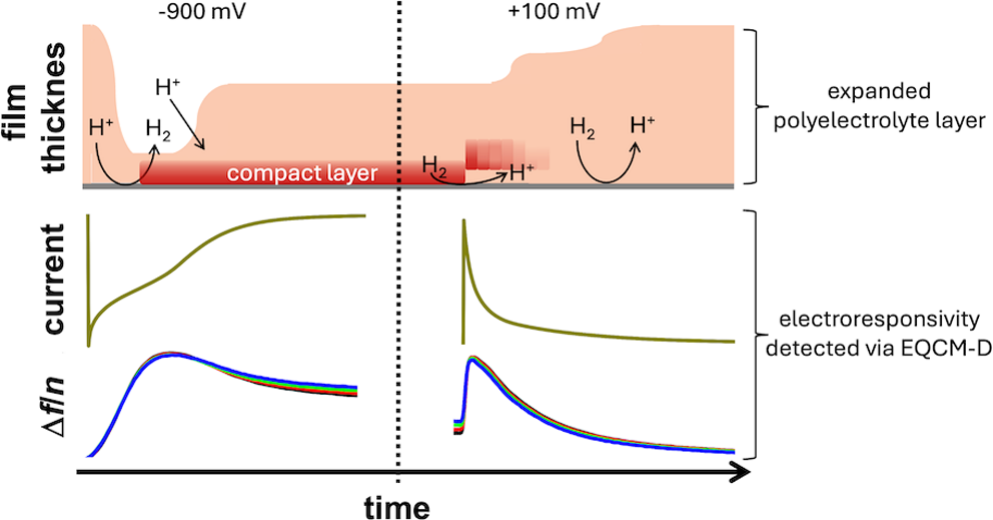

Various polycations and polyanions were sequentially adsorbed onto the gold electrode of a quartz crystal microbalance with dissipation monitoring. The study focused on determining the adsorption kinetics, viscoelastic properties, and electroresponsivity of polyelectrolyte layers. For the first time, it was demonstrated that the structure (compact or expanded) of the layers can be determined by electroresponsivity. Viscoelastic modeling alone did not provide a conclusive answer as to whether the layers were compact or expanded. The study was further enriched by streaming potential and contact angle measurements, where polyelectrolyte multilayers were formed on mica. It was found that successive adsorption of layers led to periodic inversion of the zeta potential. Systematic differences were observed between the different top layers, which were explained by intermixing between layers. The presence or absence of interpenetration, as determined by the measurements of streaming potential and contact angles, correlated well with electroresponsivity.

## 1 Introduction

### 1.1 General

Thin films of polyelectrolytes adsorbed onto solid surfaces exhibit versatile functionalities. They can act as antifouling layers, preventing protein adsorption^1^ or can reduce adhesion and friction.^2,3^ When adsorbed in expanded configuration, the layers typically exhibit responsiveness, which implies a dependence of the layer thickness on pH,^4^ temperature,^5^ ionic strength,^6^ or the electrical potential of the substrate.^7,8^ Technical advantages of electroresponsivity lie in its potential to electrically modulate surface properties.^8,9^

The behavior of surface-attached polyelectrolytes—whether weak or strong—has predominantly been investigated using brush models,^10^ which are structurally more defined than physically adsorbed chains. Despite being less well-defined, dilute adsorbates share some similarities with brushes. When this occurs, they are referred to as “pseudobrushes.”^11^ In this study, we consider the single layers of adsorbed hydrocolloids to be pseudobrushes.

**Figure 1:**
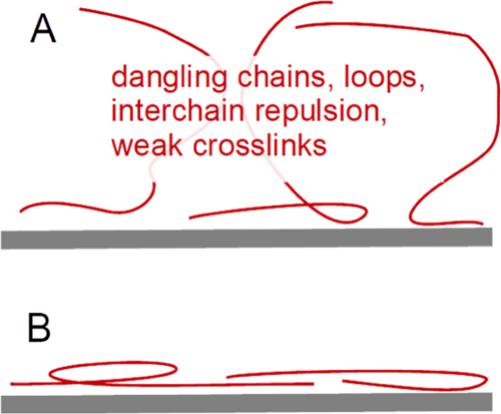
Hydrocolloids typically attach to solid surfaces in an expanded conformation (A). In contrast, simpler polymers with charges opposite to the surface charge form compact layers (B).

Whether such layers adsorb in a compact or expanded form (Figure 1) has been extensively studied in the context of polyelectrolyte multilayer deposition.^12−14^ The process is also called layer-by-layer deposition. The overall thickness of these layers may increase in proportion to the number of layers, *N*, but may in other cases also depend exponentially on *N*.^15,16^ The latter type of layers is more open and extended, with the degree of openness increasing as multilayer deposition proceeds.

Polyelectrolyte multilayers (PEMs) have been studied with the quartz crystal microbalance (QCM) early on.^17^ The QCM remains a valuable tool for monitoring deposition and, more generally, for mass-based sensing.^18−22^ In its basic (“gravimetric”) configuration, the QCM reports a negative frequency shift, which for rigid materials is proportional to the mass per unit area of the deposit, Δ*m*. The QCM with dissipation monitoring (QCM-D) yields additional parameters, including the half-bandwidth of the resonance, ΔΓ. The shift in “dissipation”, Δ*D*, is related to the shift in half-bandwidth as Δ*D* = 2ΔΓ/*f*_res_. ΔΓ supplies nongravimetric information, often utilized to infer the softness of the sample.^23,24^ The QCM-D can interrogate multiple overtones, designated by their overtone order, *n* (typically *n* = 3,5,7,9,11). For rigid layers, the values of Δ*f*/*n* agree across overtones; discrepancies indicate nongravimetric effects, paralleling instances where ΔΓ/*n* is nonzero.^25,26^ Nongravimetric effects will be discussed in detail in Section 3.3

Hydrocolloids are among the substances forming dilute adsorbates. In this context, the term “hydrocolloid” refers to polymers that are soluble in water but can form physical cross-links, enabling gel formation.^27^ The physical cross-links stabilize extended adsorbates. Due to their biocompatibility, hydrocolloids play a role in the food industry^28^ and in healthcare.^29^ They are used for rheology modification,^30^ encapsulation,^29^ wound dressing,^31^ and food coating.^32^ Hydrocolloids may be either charged or uncharged, with examples of charged hydrocolloids including chitosan (Chit), alginate, and various types of carrageenans (Carrs). Positively charged Chit is a widely utilized linear polysaccharide derived from the deacetylation of chitin, sourced from the exoskeletons of crustaceans (e.g., crabs and shrimp) as well as insects and fungi.^33^ In acidic environments, the amino groups of Chit become protonated, resulting in pH-responsivity. Carrs are negatively charged polysaccharides comprising different subtypes.^28,34−36^ Kappa-carrageenan (κ-Carr) and iota-carrageenan (ι-Carr) form three-dimensional double-helix networks through cross-linking of adjacent sulfate groups, while the sulfate groups of lambda-carrageenan (λ-Carr) do not cross-link and thus do not form gels.^37^ The mechanical properties of Carr-based films correlate with their capacity to swell in mucous tissue.^38^

Kara et al. employed QCM-D to investigate how variations in salt concentration and the addition of water-soluble graphene oxide affect the formation of Carr/Chit multilayers.^39^ They find that Na^+^ ions and hydrophilic graphene oxide sheets enhanced the deposited mass and the viscoelasticity (i.e., the softness). In another QCM-D study, Oliveira et al. demonstrated that the structure of Carr/Chit films is influenced by salt concentration, pH, and Carr type.^40^

In this study, we expand upon QCM-based investigations of PEMs by examining electroresponsivity. To the best of our knowledge, electroresponsivity has been applied for the first time to study the structure (compact or expanded) of polyelectrolyte multilayers. Until now, the degree of swelling of polyelectrolyte multilayers has primarily been determined using classical gravimetric measurements (via QCM-D). More specifically, we investigate how variations in electrode potential influence the sets {Δ*f*/*n*, ΔΓ/*n*}. A second novel aspect was establishing correlations between electroresponsivity, streaming potential, and contact angles. While electrically modulating the properties of these surfaces is attractive in applications,^7^ the utility of electroresponsivity as a characterization tool is equally important.

The experiment employs an electrochemical QCM-D (EQCM-D),^41^ which is well-established in the field. Unique features of the experiment include a time resolution of 2 ms as well as accumulation and averaging, which enhance frequency precision to a few millihertz. Voltage modulation influences the local pH and the concentrations of co-ions and counterions near the electrode surface. For simple electrolytes, these ions constitute the electric-double layer, which comprises rigidly adsorbed ions exhibiting slow dynamics (the “Helmholtz layer”) and a cloud of detached ions with rapid dynamics (the “diffuse double layer”). Simple electrolytes cause a rapid QCM response upon voltage modulation, a phenomenon attributable to the recharging of the diffuse double layer. However, in the presence of zwitterions or proteins, the situation becomes more complex,^42^ because voltage modulation induces a second, slower process. Adsorbed polyelectrolyte layers further complicate the system.

In the first step, cationic polyelectrolytes were adsorbed onto the resonator surface in the form of branched poly(ethylenimine) (bPEI), poly(diallyldimethylammonium chloride) (pDADMAC), or chitosan (Chit). While Chit is generally classified as a hydrocolloid, pDADMAC typically is not. Although bPEI is not traditionally categorized as a hydrocolloid, it exhibited similar behavior in this study. Both Chit and bPEI are weak polyelectrolytes, possessing a net charge only at low pH. In a second step, polyanions—either carrageenan (Carr) or polystyrenesulfonate (PSS)—were adsorbed onto the preadsorbed positively charged layers. Most forms of Carrs, such as κ-Carr and ι-Carr, are classified as hydrocolloids, whereas PSS is not.

All monolayers and bilayers were evaluated for their electroresponsivity. We argue that these findings contribute to a deeper understanding of the dynamics and responsiveness of these layers, as well as their structural characteristics. Concurrent experiments involved the formation of analogous PEMs on mica. The zeta potentials of subsequent layers were evaluated using streaming potential measurements (see Section 3.6). Additionally, the contact angles, which are important indicators of wettability, of the PEMs were determined (see Section 3.7).

### 1.2 Advantages of QCM-D for Investigating Soft Layers at Interfaces

Ref (26) discussed in some depth the advantages of quartz crystal microbalance with dissipation (QCM-D) in studies of soft layers at interfaces. The following paragraphs recapitulate a few essentials. The distinctive features of QCM-D are best elucidated by a comparison with optical reflectometry, particularly surface plasmon resonance spectroscopy (SPR). SPR shares several features with QCM-D. QCM-D can be understood as a form of shear-wave reflectometry (see eq 36 in ref (26)).

In scenarios where the height of the sample is much below the wavelength of either light or acoustic shear waves, reflectivity becomes proportional to the integral of a contrast function. For SPR, the contrast function is (*n*^2^(*z*) – *n*_bulk_^2^)/*n*^2^(*z*), whereas for QCM-D, it is (η̃(*z*) – η_bulk_)/η̃ (*z*). The tilde indicates of complex-valued parameter. Although the underlying mathematics is similar, the values to be inserted into the contrast functions differ.

The refractive index profile in optical techniques, denoted as *n*(*z*), is influenced primarily by electronic polarizability with little dependence on conformation and dynamics. Moreover, *n*(*z*) deviates from *n*_bulk_ by only a few percent, with this difference correlating with the polymer volume fraction. When a sample expands, the contrast function diminishes in proportion to the increase in thickness. These two factors tend to counterbalance each other, allowing optical reflectometry to predominantly determine the adsorbed amount, largely independent of swelling or dynamics.

In contrast, the viscosity in the contrast function probed by the QCM-D is highly sensitive to chain conformation. Additionally, |η̃(*z*)| generally exceeds η_bulk_ by easily a factor 10, leading to a contrast function that approaches unity within the larger part of the adsorbate. The lack of compensatory effects between increased thickness and reduced contrast function—as seen in optical methods—renders QCM-D considerably more responsive to swelling and, more broadly, to conformation than optical reflectometry.

Two more distinctions are noteworthy:

- Optical absorption is typically nonessential in reflectometry, wherein SPR primarily infers the adsorbed amount from shifts in the in-plane wavevector, which for most practical purposes is a real-valued parameter. In contrast, all soft samples analyzed via QCM-D exhibit viscoelastic behavior, where the real and imaginary components of η̃ are equally significant. The resonance bandwidth (equivalent to the “dissipation”, *D*) conveys information about as relevant as the frequency shift.
- The complex viscosity depends on frequency (that is, on overtone order) whenever the sample exhibits relaxation dynamics on time scales comparable to the inverse resonance frequency. This phenomenon, referred to as “viscoelastic dispersion”, provides insight into relaxation processes.

### 1.3 Voltage Modulation as a Novel Mode of Probing Samples by QCM-D

Generally speaking, the depth of information provided by the QCM-D is better than that provided by SPR, because the number of experimentally derived parameters (Δ*f*/*n* and ΔΓ/*n* on a few overtones) is larger. However, this advantage is not always fully realized due to the challenge of fitting viscoelastic models to the QCM-D results. The modeling process often yields an array of solutions, which all match the data equally well (see Section 1.4). A novelty of this work, which has potential beyond hydrocolloids, lies in combining QCM-D measurements with electromodulation.

Electromodulation applied to QCM-D bears conceptual similarity to electroreflectance in optics. The optical reflectivity of a conductive surface immersed in a liquid is contingent upon the electrical potential applied to that surface.^43−45^ Complications arise in optics due to the dual contributions to electroreflectance: one from the solid substrate and another from the electric-double layer. The authors of ref (46) developed methodologies to distinguish between these contributions and also transformed the optical reflectometer into a modulation microscope.

We show that the determination of structural properties—an endeavor difficult to accomplish using only the conventional sets of Δ*f*/*n* and ΔΓ/*n*—becomes feasible when the structure is perturbed by modulating the substrate potential. To summarize a more intricate argument, dilute layers respond to electromodulation more strongly than compact layers. Currently, no standardized protocol exists that can be applied generically across all QCM-D experiments involving electromodulation. Developing such a protocol is left for future research. Presumably, it would entail the application of small increments in surface potential (dϕ), with analytical approaches based on the derivatives d(Δ*f*/*n*)/dϕ and d(ΔΓ/*n*)/dϕ, as well as the time constants of the response. Time constants will be meaningful whenever the response consists of an exponential approach to a new steady state.

In this study, we apply larger potential steps and find that the response is different from approaches to a new state with well-defined time constants. Consequently, interpretation must occur in light of the specific characteristics of these samples. Nonetheless, we claim that electromodulation—technically straightforward to implement—will make the QCM-D more versatile and more capable of offering insights into the structure and dynamics of soft materials.

Electromodulation has previously been explored by the Paris group using what the group calls “AC-electrogravimetry”.^47^ These experiments were carried out in the frequency domain by applying a small sinusoidal potential perturbation similar to electrochemical impedance spectroscopy (EIS) and slowly sweeping frequency. Using an oscillator circuit, resonance frequency changes of the fundamental mode were tracked. Information beyond gravimetry is not amenable as neither a dependence on overtone order nor bandwidth information are available. The time-domain version of electromodulation (used here) has previously been applied in studies of the diffuse double layer^48^ and of electrochemical reactions.^49^ Its application to hydrocolloid layers is novel.

Given that hydrocolloids are weak polyelectrolytes with charge states influenced by pH, and given that the near-surface pH depends on electrode potential, the pH plays a special role in these electromodulation experiments. Furthermore, the influence of interdigitation between layers on electroresponsivity highlights the materials aspects inherent in this work.

### 1.4 Distinction Between Compact and Expanded Layers Based on Viscoelastic Modeling and Electromodulation

In this section, we provide background on how the results obtained with the QCM-D should be interpreted. A QCM response with ΔΓ/*n* ≪ −Δ*f*/*n* and Δ*f*/*n* independent of *n* is referred to as “Sauerbrey-type behavior” in the following. According to the Sauerbrey model, such a “gravimetric” response is caused by rigid layers. However, a Sauerbrey-type response is also obtained when the sample consists of a thin, liquid-like layer.^50^ This possibility was overlooked in the early QCM-based studies of layer-by-layer deposition. In this section, we approximate the sample as a single layer with a sharp interface to the bulk. The function *G̃*(*z*) with *G̃* = iωη̃ the complex shear modulus then amounts to a box profile. The arguments outlined below are not limited to box profiles. They only require thin layers, meaning that the profile extends to a distance much smaller than the penetration of the shear wave, δ = (2η_bulk_/(ρω))^1/2^. δ is between 100 and 200 nm for 5 MHz resonators in water, depending on overtone order.

For thin layers, the more general equation, which predicts Δ*f* and ΔΓ as a function of the thickness, *d*_f_, and the layer’s shear modulus, *G̃*_f_, can be Taylor-expanded in thickness.^26,51^ The resulting, simplified equation is
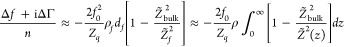
1*f*_0_ is the frequency of the fundamental (*f*_0_ = 5 MHz, here), *Z*_q_ = 8.8 × 10^6^ kg/(m^2^s) is the shear-wave impedance of the resonator plate, ρ_f_ is the density of the film, *d*_f_ is the thickness of the film, the subscript *f* denotes the layer (the film), and  is the shear-wave impedance. The tilde denotes a complex parameter. The contrast function (in square brackets) accounts for viscoelasticity. In the second step, the product of the film thickness and the contrast function was replaced by an integral of the contrast function over the distance from the surface. *Z̃*_f_^2^ then becomes a function of distance from the surface, *Z̃*^2^ (*z*). For simplicity, constant density (ρ the same in the film and in the bulk) was assumed. The formulation in terms on an integral was intended to emphasize that the following argument holds for continuous viscoelastic profiles as well. We revert to films with a sharp upper boundary in the following. We argue that modeling layers as films in this sense captures the essence of electroresponsivity. The equivalent thickness of an adsorbate with no sharp boundary is calculated from the first moment of the contrast function (Section 4.6.2 in ref (26).).

For rigid samples, the second term in the contrast function vanishes and the Sauerbrey case is recovered. If the layer is stiff but still not perfectly rigid, the second term in the contrast function is small. It amounts to a viscoelastic correction, which can be quantitatively analyzed rather easily.^26^ The problem occurs when the layer is liquid-like with a Newtonian viscosity, η_f_, slightly larger than η_bulk_. For such layers, it is most transparent to express viscoelasticity in terms of complex viscosity because the viscosity of the layer can then be directly compared to the viscosity of the bulk. We write the viscosity as η̃_f_*=* (η_f_′ – iη_f_″) *= G̃*_f_/(iω), where the real part, η_f_′, is larger than the imaginary part, η_f_″. Expressed in terms of viscosity, the shear wave impedance is . Equation 1 turns into
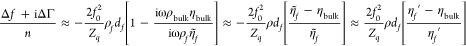
2If η′_f_ ≫ η″_f_, the contrast function is almost real and independent of *n*. Such layers look like Sauerbrey layers to the experimentalist, even though they are not at all rigid. Moreover, the thickness, *d*_f_, cannot be inferred from Δ*f*/*n* as long as the viscous contrast (the term in square brackets) is not known.

The diffuse double layers from electrochemistry exemplify the problem with such configurations, studied with an EQCM.^48^ The viscosity of the double layer changes, when counterions and co-ions are exchanged between the double layer and the bulk upon switching the electrode potential. A Sauerbrey-type response is seen even though the diffuse double layer is liquid-like. The thickness of the layer with altered viscosity cannot be inferred from the QCM response. A caveat: A Sauerbrey-type response might also be caused by a changed thickness of the Helmholtz layer, which consists of rigidly adsorbed ions. It is argued in ref (48) that the two cases (changed properties of the diffuse double layer or changed properties of the Helmholtz layer) can be distinguished based on the kinetics of the response (fast for the diffuse double layer, slower for the Helmholtz layer).

The same problem occurs for polyelectrolyte layers (hydrocolloids or conventional). When the sets of parameters {Δ*f*/*n*, ΔΓ/*n*} are used as input to viscoelastic modeling,^50^ the χ^2^-landscapes as a function of layer thickness display shallow minima and sometimes even contain two separate minima, one for the compact and one for the extended form. Viscoelastic modeling starting from the sets of {Δ*f*/*n*, ΔΓ/*n*} alone does not allow to distinguish between the compact and the expanded form. Because the χ^2^-landscape is shallow, configurations intermediate between “compact” and “expanded” are also possible (Section 3.2).

Further information on the structure of the adsorbate can be obtained by perturbing it. The approach is classical in nature. One example is the study of the Zeeman effect in vibrational spectroscopy, which aids in the assignment of certain lines to certain transitions.^52^ Another example, closer to this work, is electric-double-layer-modulation microscopy as reported in ref (46). In this study, the authors exploit electroreflectance, which is the optical analog of AC-electrogravimetry.^47^

Modulating the surface potential in QCM-D experiments follows the same strategy. The reflectivity of acoustic shear waves at the resonator surface depends on the electrical surface potential in a way that has many analogies to electroreflectance. The QCM can be viewed as a shear wave reflectometer (eq 36 in ref (26).). Unlike AC-electrogravimetry, as introduced by the Paris group in the 1990s,^47^ the experiments described below (and also reported in refs (42) and (48)) are conducted in the time domain and cover nongravimetric effects. Electromodulation, as carried out here, is not particularly demanding with regard to equipment or procedures. It may well become a routine characterization tool.

## 2 Experimental Section

### 2.1 Materials and Preparation

Ultrapure water (Milli-Q Elix & Simplicity 185 purification system, supplied by Millipore SAS France) and sodium chloride, NaCl (analytical grade, pure p.a., Avantor Performance Materials Poland S.A) were used for the preparation of 0.01 M NaCl solutions with a pH of 5.8. Hydrochloric acid, HCl, (36.5–38.0%, Merck KGaA, Germany) was used without further purification for the preparation of solutions with an ionic strength of 0.01 M and a pH 2.

High-quality muscovite (mica, supplied by Continental Trade Ltd., Poland) was used as the substrate in measurements of the streaming potential and wettability. Ti/Au 5 MHz quartz sensors, hereafter referred to as gold substrates, were purchased from QuartzPro AB, Sweden.

Aqueous solutions of bPEI (500 mg/mL) with a molecular mass of 70 kDa were obtained from Polysciences Europe GmbH (Germany). pDADMAC with a molecular mass of 101 kDa and PSS with a molecular mass of 70 kDa were supplied by PSS Polymer Standards Service GmbH (Germany). Chit with a molecular mass of 50–190 kDa^53^ and Carr with a molecular mass of 475 kDa^54^ were obtained from Sigma-Aldrich (Germany). All polyelectrolytes were used as received. A detailed characterization of Carr is provided in the Supporting Information.

All polyelectrolyte solutions, other than the Chit solution, were prepared by dissolving the appropriate amount of the polyelectrolyte in a 0.01 M NaCl solution with pH 5.8, resulting in a polyelectrolyte concentration of 100 mg L^–1^. Before adsorption, the solutions were diluted to a concentration of 5 mg L^–1^ with 0.01 M NaCl. Chit was treated differently, as it requires an acidic pH to be well-soluble. Stock solutions of Chit (bulk concentration: 100 mg L^–1^) were prepared by dissolving the suitable amount of powder in 0.01 M HCl with a pH of 2.0.

The pH after dissolution was 5.8 for all samples except bPEI and Chit. In these cases, the pH was 7.45 and 4.0, respectively. The bPEI solutions were slightly basic because the amine groups are partly protonated in 0.01 M NaCl.

The temperature was *T* = 298 K in all experiments.

### 2.2 Adsorption Monitoring with the QCM-D

Layer formation was monitored with a QCM-D (E1, Q-sense, Sweden), using a flow rate of 1.33 × 10^–3^ mL s^–1^. Solutions of 5 mg L^–1^ polyelectrolyte (Carr, pDADMAC, bPEI, Chit, PSS) were prepared as described in Section 2.1. Since the substrate carries negative charge, the anchor layers (bPEI, pDADMAC, Chit) must consist of polycations. First, the anchor layer was adsorbed onto 14 mm gold-coated quartz resonators (Ti/Au) with a fundamental frequency of 5 MHz (QuartzPro, Sweden) for 30 min, followed by a rinse in a compatible electrolyte (0.01 M NaCl, pH 5.8) for another 30 min. In some experiments, a 5 mg L^–1^ solution of polyanions (Carr or PSS) in 0.01 M NaCl at pH 5.8 was introduced into the QCM cell as a second step. The adsorption onto the first layer was monitored for 30 min, followed by a second rinse. In a third step, the coated resonator was removed from the Q-sense cell, dried with nitrogen, and transferred to another EQCM cell for the study of electrogravimetry.

### 2.3 Electromodulation and Electroresponsivity

Swelling and deswelling upon variation of the electric potential was studied in a home-built EQCM cell containing 1 mL of 0.01 M NaCl as the supporting electrolyte. Because the resonators needed to be transferred from the measurement cell used for monitoring adsorption to a second cell for electromodulation, the reference frequencies of the bare resonators were no longer available. Resonance frequencies always change by a few Hertz during dismounting and remounting due to variation in static stress.^55^ The frequency shifts induced by the variable surface potential could therefore not be referenced to the frequencies of the bare crystals.

The coated front electrode of the resonator (electrode area: 1.17 cm^2^) served as the working electrode in a three-electrode setup. Platinum wires acted as both the pseudoreference electrode and the counter electrode. The potential at the resonator’s front electrode was switched between +100 and −900 mV versus Pt using the potentiostat Interface 1010E (Gamry). These voltages induced a capacitive current (leading to double layer recharging) as well as a Faraday current. The Faraday current may involve the hydrogen evolution reaction (HER) and its inverse, as well as the oxygen reduction reaction (ORR) and its inverse. In both cases, positive potentials lower the pH, while negative potentials increase it.

The resonances were probed using a multifrequency lockin amplifier (MLA) supplied by Intermodulation Products AB (Sweden), employing a frequency comb with up to 32 frequencies simultaneously applied to the resonator. For more information on the MLA-driven fast EQCM, see ref (48). The frequency spacing in the comb was 500 Hz, enabling a time resolution of 2 ms. Frequency shifts, Δ*f*(*t*), and shifts in the half-bandwidth, ΔΓ(*t*), were acquired on four overtones (*n* = 3,5,7,9) at 15, 25, 35, and 45 MHz. Here, “Δ” denotes shifts relative to the frequencies and bandwidths immediately preceding the switching event. Because this state is not a well-defined reference state, interpretation must proceed without explicit quantitative modeling.

### 2.4 Viscoelastic Modeling

The modeling was performed using the software package PyQTM.^50,56^ The underlying equation is^57−59^
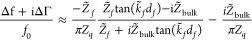
3 is the wavenumber;  is the shear wave impedance. The subscripts “f” and “q” denote the film and the resonator (the quartz resonator), respectively. Eq 3 applies to all thicknesses, at least in principle. (eq 2 results from a Taylor expansion of eq 3 in thickness, *d*_f_.) Eq 3 does, however, make use of the small-load approximation.^26^

The shear modulus, *G̃*, was expressed as iωη̃ = iω(η′ – iη″). Soft matter displays viscoelastic dispersion in the MHz range, meaning that η′ and η″ depend on frequency. Because the frequency range covered by the QCM is small, the frequency dependence of η′ and η′′ can be approximated with power laws of the form^26^
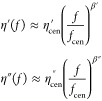
4The subscript *cen* denotes a frequency in the center of the frequency range covered by the QCM (typically *f*_cen_ = 35 MHz). The power law exponents, β′ and β″, are constrained by the Kramers–Kronig relations. One has −2 ≤ β′ ≤ 0 and −1 ≤ β′′ ≤ 1 if the complex viscosity, η̃, is used as the parameter quantifying viscoelasticity. The model has 5 free parameters, which leads to problems with the uniqueness of fitting results. More details on modeling and, also, on the ambiguities in interpretation are provided in ref (50).

### 2.5 Streaming Potential Measurements

Measurements of the streaming potential were performed to determine the ζ-potential of the top layer. Freshly cleaved mica sheets, prepared before each experiment, were used as substrates. Mica replaced gold for two reasons. First, streaming potential measurements can be influenced by the bulk conductivity of the gold substrate.^60^ Second, the streaming potential of an electron-conducting substrate is a nonlinear function of flow rate.^61^

The streaming potential was measured using a pair of Ag/AgCl electrodes positioned before and after a parallel-plate channel formed by two mica sheets, separated by a Teflon gasket, following the procedures from ref (62). The electrolyte was a 0.01 M NaCl solution with pH 5.8. Four different pressure differences were applied. Multilayers were formed inside the cell. The ζ-potential was derived from the dependence of the streaming potential on the pressure difference using the Smoluchowski equation.^63^ While the QCM measurements were limited to the first two layers, streaming potential measurements occurred on samples with up to eight layers, ensuring that the influence of the mica substrate became negligible.^13,64^

### 2.6 Contact Angle Measurements

The contact angles on the substrates (mica or gold) and on the polyelectrolyte layers were determined using the sessile drop method in static mode. Pure mica sheets were entirely hydrophilic, with a nominal contact angle of zero, while the reference contact angle for gold was 98°. Measurements were carried out using the Kruss DSA100 apparatus, where a single drop of ultrapure water (2 μL) was deposited.^65^ Ten measurements were taken at different locations for each sample. Contact angles were calculated using KRUSS Drop Shape Analysis Software (DSA4). The tangent method was applied, fitting a polynomial to the drop profile near the meniscus.^65^

## 3 Results

### 3.1 Adsorption Kinetics

**Figure 2:**
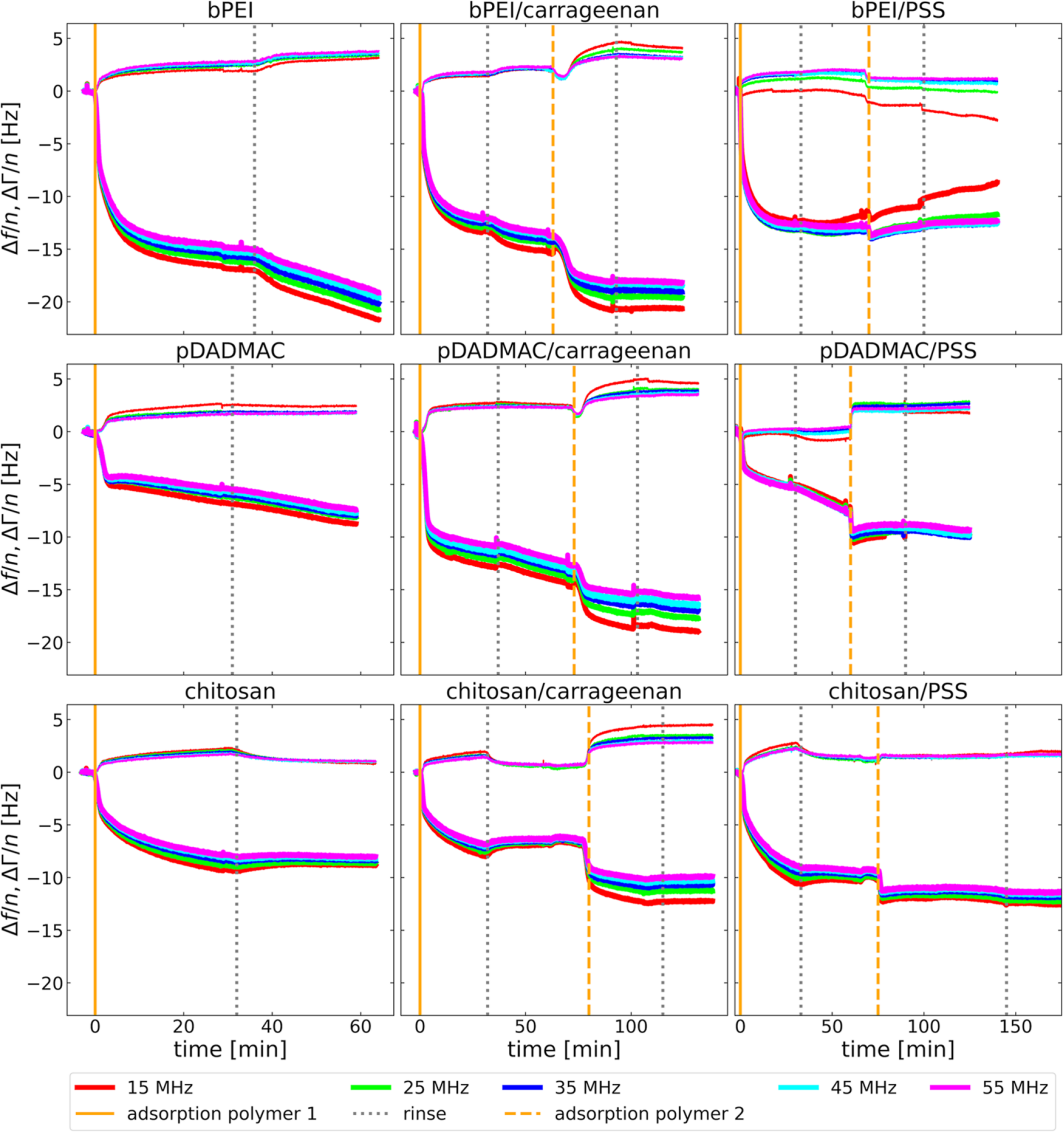
Shifts in overtone-normalized resonance frequency, Δ*f*/*n*, and half-bandwidth, ΔΓ/*n*, upon adsorption of different mono- and bilayer systems. The left column shows the formation of a polycation monolayer composed of bPEI, pDADMAC, or Chit (first, second, and third row, respectively). The middle column shows the formation of a bilayer composed of one of the polycations and Carr as polyanion on top. The right column also shows bilayers, where PSS is the top layer. Orange vertical lines indicate the start of adsorption, while the gray vertical lines indicate the start of rinsing.

Figure 2 shows the adsorption kinetics in terms of Δ*f*/*n* and ΔΓ/*n* on five overtones (*n* = 3,5,7,9,11). Because gold is negatively charged at pH 5.8, the first layer to adsorb must be cationic (bPEI, pDADMAC, or Chit). These experiments are shown in the left column. Adsorption was started at the times indicated by a vertical orange line and was followed by rinsing, marked by a dotted gray line. The electric potential of the gold surface was not actively controlled during adsorption.

During monolayer adsorption, Δ*f*/*n* decreases while ΔΓ/*n* slightly increases. This behavior is commonly attributed to the formation of stiff layers, similar to Sauerbrey films. The rinsing does not significantly alter Δ*f*/*n* and ΔΓ/*n*, indicating minimal desorption. Additionally, the viscosity of the bulk polyelectrolyte solution (which differs slightly from the buffer viscosity used during rinsing) had a negligible effect. This result is expected, given the low concentration of the polyelectrolyte in the bulk (5 mg L^–1^).

In a second step, negatively charged Carr (middle column) and negatively charged PSS (right column) were adsorbed onto the cationic monolayers. The start of the second adsorption step is indicated by a dashed orange vertical line, followed by a second rinse indicated by a dotted gray line. Except for the bPEI/PSS system, Δ*f*/*n* further decreased and ΔΓ/*n* further increased upon adsorption of the second layer. The second rinsing step had little effect on Δ*f*/*n* and ΔΓ/*n*.

The differences in adsorption kinetics can be attributed to variations in the chemical interactions between the polyelectrolyte and the gold substrate or the first polyelectrolyte layer. These differences are outside the scope of this study. Interpretation would require more repetitions of the layer-formation experiments. Minor variations due to drift, equilibration, imperfect temperature control, and liquid flow are typical for such experiments.

### 3.2 Ambiguity in Interpretation

**Figure 3:**
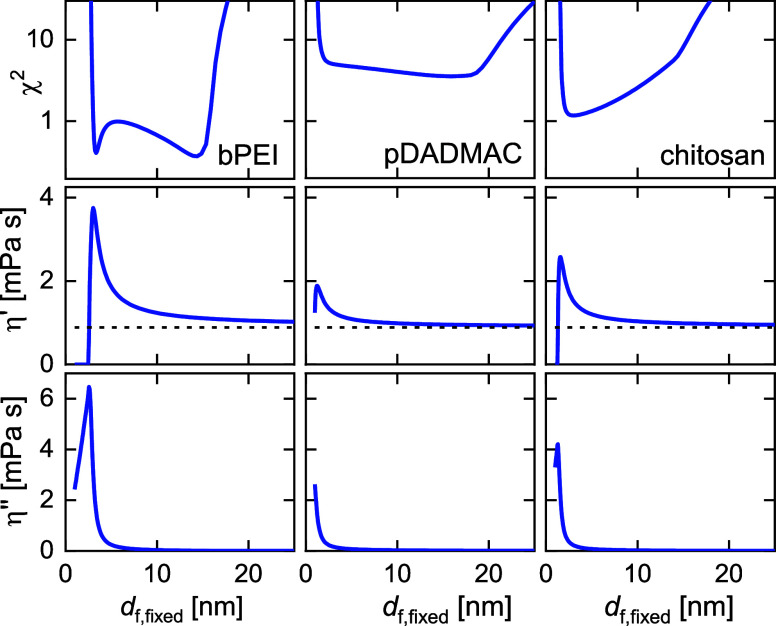
Top: χ^2^-landscapes at *t* = 16 min, obtained by fitting the viscoelastic model as described in Section 2.4 to the sets {Δ*f*/*n*, ΔΓ/*n*}. The thickness was fixed to the values indicated on the *x*-axis, leaving the remaining parameters (η′_f_, η″_f_, β′, and β″) free for χ^2^-minimization.

While one might be tempted to compute the layer thickness from the decrease in frequency observed upon adsorption using the Sauerbrey equation, such a conversion is problematic because the layers are very soft. Figure 3 shows χ^2^-landscapes obtained from fits with PyQTM^56^ for the monolayers (bPEI, pDADMAC, Chit). These landscapes were calculated for a single data point at time *t* = 16 min. The thickness was not a free parameter, but rather was fixed to the values shown on the *x*-axis. The fitting algorithm used the remaining model parameters (η′_f_, η″_f_, β′, and β″) to minimize χ^2^. The χ^2^-landscapes as a function of thickness are rather flat, as usual for such thin layers.^50^ In the case of bPEI, there are two distinct minima in the function χ^2^(*d*_f_). These correspond to a stiff layer and a liquid-like layer, as can be inferred from the values of η′_f_ and η″_f_, shown in the center and at the bottom. For the expanded layers, η′_f_ is slightly higher than the viscosity of water and η″_f_ is close to zero. In all cases, layer conformations intermediate between compact and expanded are compatible with the χ^2^-landscapes, as well. Figure S2 in the Supporting Information shows two sets of fits to the adsorption kinetics of bPEI. The guess values for fitting were set to 2.5 nm (compact) and to 12 nm (expanded). These are values close to the two local minima on the upper left in Figure 3. The fit finds the two local minima corresponding to these two guesses for all times. Depending on whether the χ^2^-minimization was started from the compact or the extended conformation, two rather different sets of derived parameters describing the adsorption kinetics were obtained.

As the top row shows, the model has difficulties distinguishing between the compact conformation (thickness: a few nm) and the extended conformation (thickness: >10 nm). The elastic component of the viscosity, η″, is high for the compact conformation and low for the extended conformation (bottom row). The dashed horizontal lines denote the viscosity of water. The parameters β′ and β′′ also depend on *d*_f,fixed_ (not shown).

### 3.3 Potential-Dependent Swelling and Deswelling (Electroresponsivity)

It is challenging to determine how expanded the adsorbed polyelectrolytes are solely based on sets of {Δ*f*/*n*, ΔΓ/*n*}. However, electroresponsivity clearly indicates an expanded state when the substrate potential is sufficiently positive.

**Figure 4:**
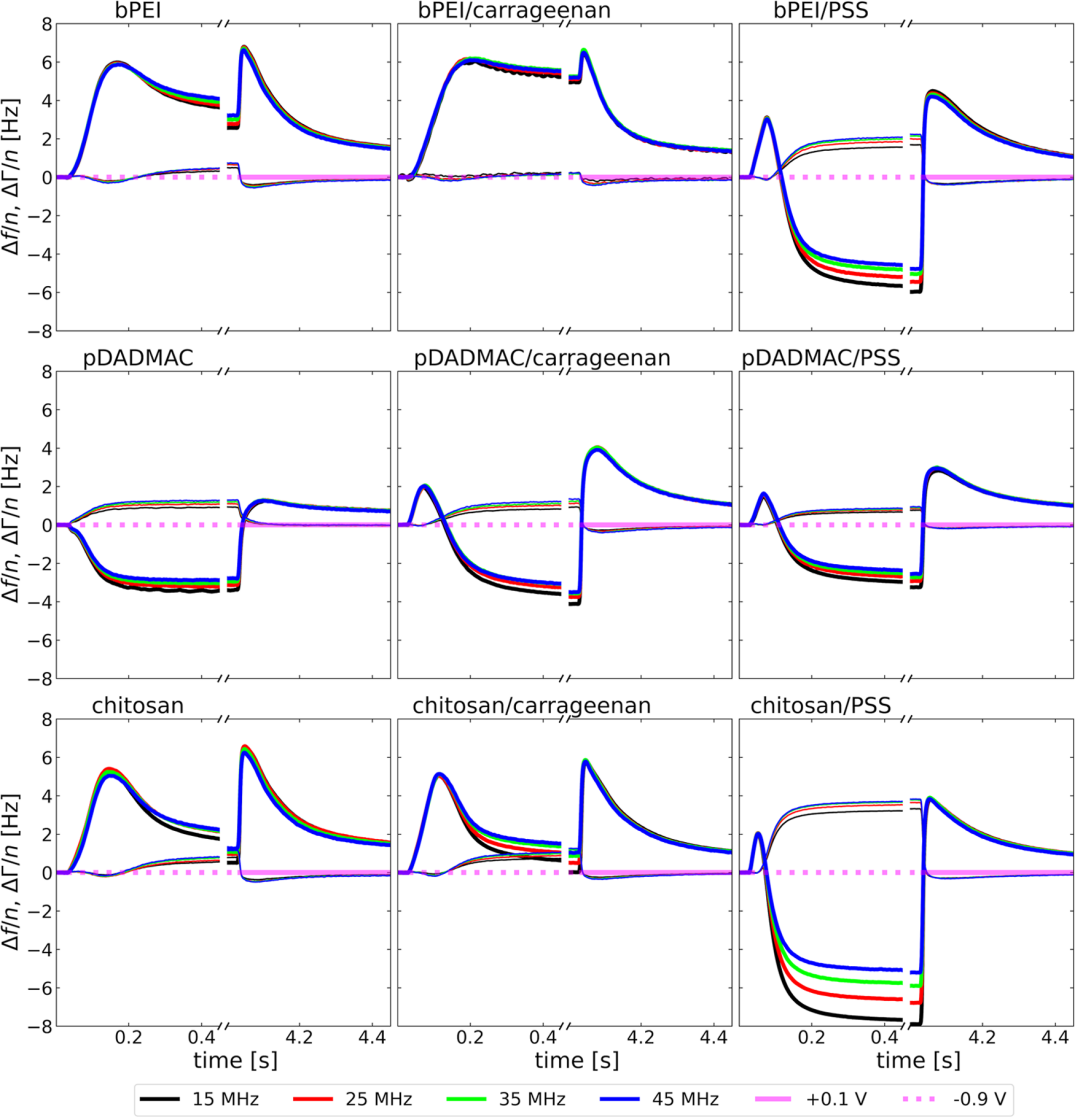
Changes in Δ*f*/*n* (thick) and ΔΓ/*n* (thin) versus time. The horizontal line in magenta (full or dashed) indicates the electrode potential. Δ*f*/*n* and ΔΓ/*n* are referenced to the values immediately before switching to negative voltages.

Figure 4 shows the changes in Δ*f*/*n* and ΔΓ/*n* following voltage steps between −0.9 and +0.1 V vs Pt. For clarity, the time span was limited to 400 ms after each step, within a total modulation period of 10 s. The electrode potential is indicated with a solid or dashed horizontal magenta line. The thick and thin lines show Δ*f*/*n* and ΔΓ/*n*, respectively. Both Δ*f*/*n* and ΔΓ/*n* are referenced to the values immediately before switching the potential to negative. It should be noted that this is not a well-defined reference state for a viscoelastic analysis. Presumably, it is a rather compact state (Section 3.4). A good reference state would be the bare crystal. Unfortunately, these frequencies are no longer available after transferring the resonator from the first measurement cell, where adsorption was monitored, to the second cell, used to study electromodulation (see also Section 2.3).

For all systems except the pDADMAC monolayer, the kinetics exhibits two processes. In the first process, Δ*f*/*n* increases while ΔΓ/*n* decreases. Such a finding is typically interpreted as a contraction. The second process, characterized by a decrease in Δ*f*/*n* and an increase in ΔΓ/*n*, corresponds to an expansion. The directions of the effects (contraction or expansion) are independent of the direction of the voltage step. The pDADMAC monolayer is the one exception to this behavior. Unlike hydrocolloids, pDADMAC is expected to adsorb in a compact form. It exhibits only one process, the sign of which depends on the direction of the voltage step.

Neither of these processes can be attributed to recharging of the diffuse double layer, as they do not change sign alongside the voltage step. Furthermore, double layer recharging should have occurred on the millisecond time scale,^42,48^ whereas the processes observed here last for tens of milliseconds.

### 3.4 Electric Currents

**Figure 5:**
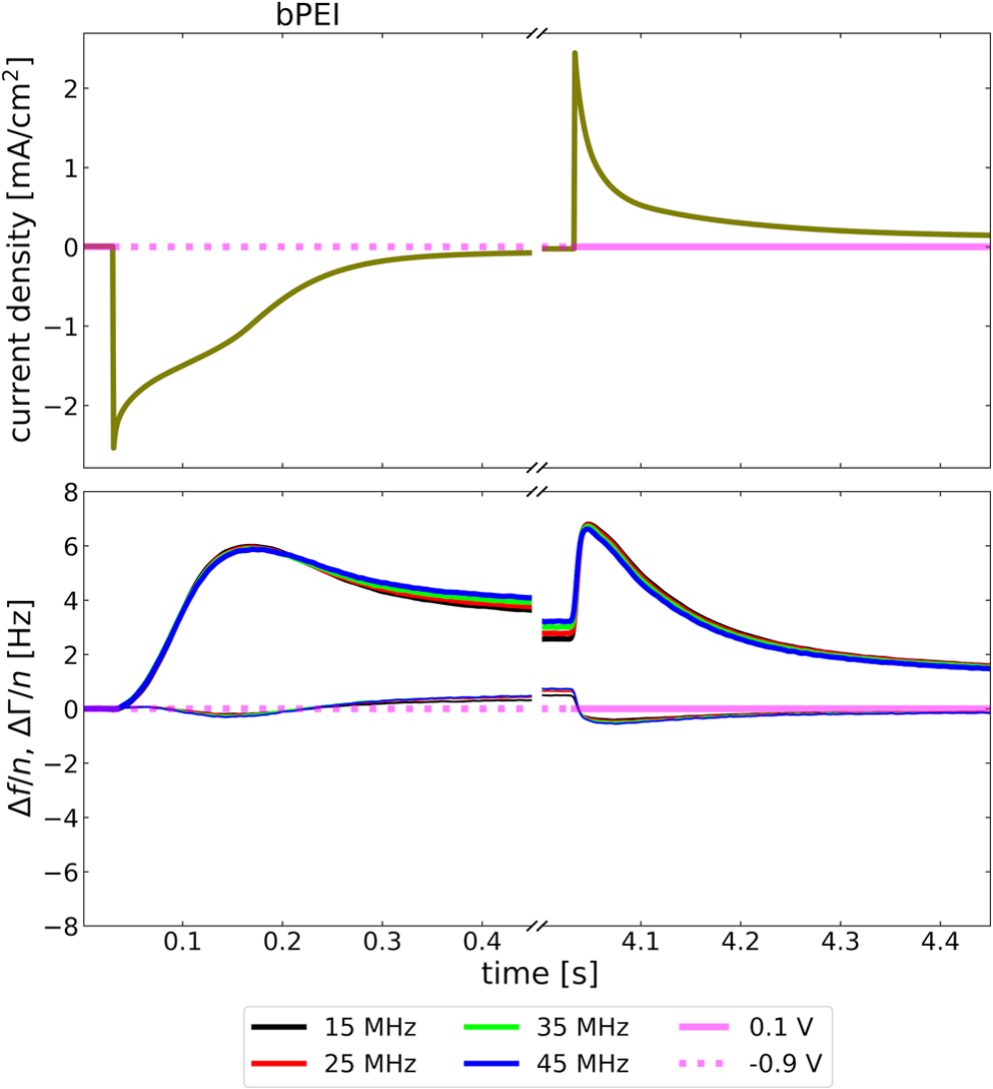
There is an asymmetry in the shape of the current trace after switching the electrode potential, which can be explained with collapsed layers forming a barrier against the transport of H^+^.

Figure 5 shows the electrical current together with the QCM response for bPEI. Similar figures for the other layer systems are provided in Figure S3 in the Supporting Information. An asymmetry is observed between the two directions of the voltage step. This asymmetry is most plausibly explained by pH effects and a collapsed adsorbate forming a barrier to the transport of H^+^.

**Figure 6:**
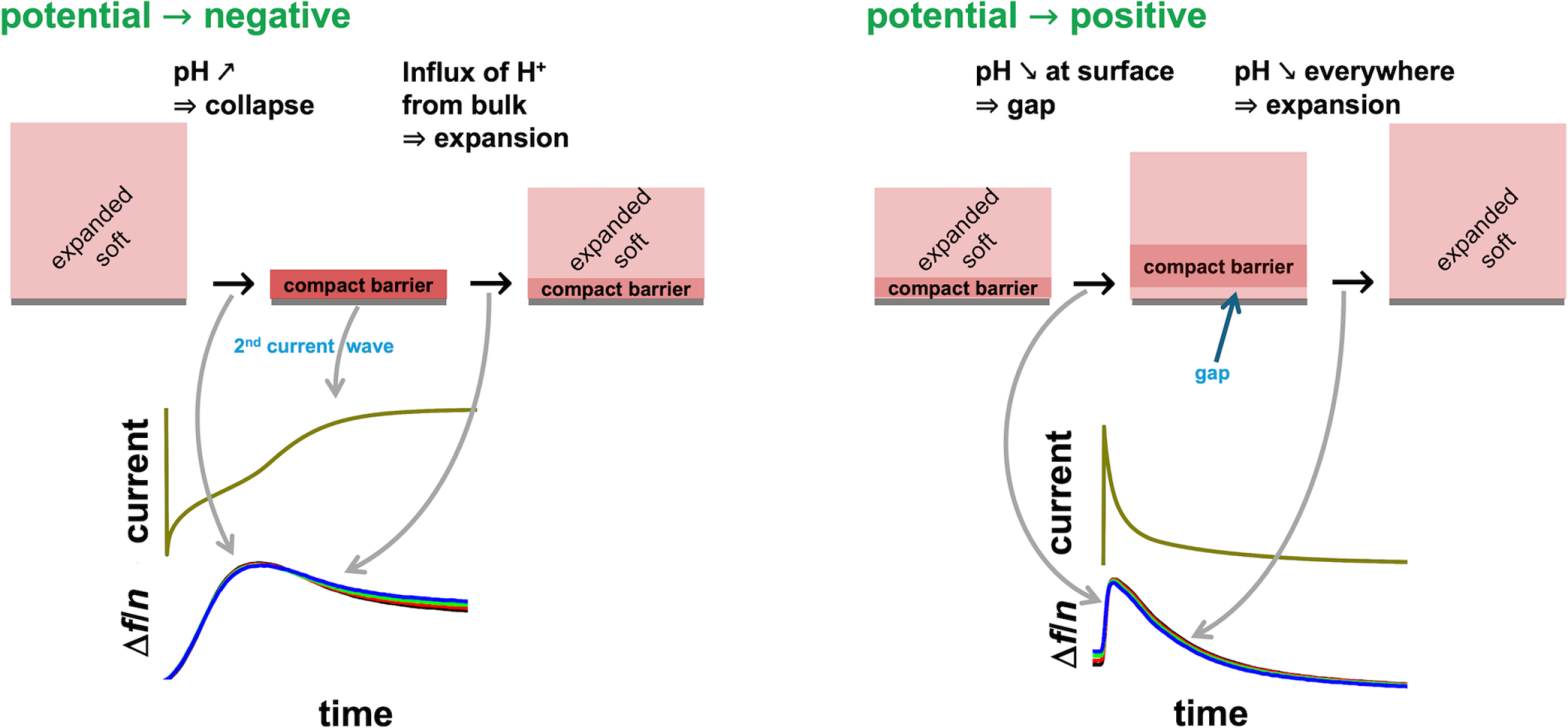
Sketch of mechanisms, which may lead to an initial increase in frequency after switching the voltage (regardless of the direction of the switching). The explanation hinges on pH and a compact barrier.

When the electrode potential is switched to negative (left in Figure 6), the pH increases due to H^+^ consumption in the hydrogen evolution reaction at the electrode. (The oxygen reduction reaction would also increase the pH.) The increased pH causes Chit and PEI to collapse, which is reflected in an increased frequency. Because the collapsed layer blocks the flow of H^+^ to the electrode, H^+^ flowing in from the bulk decreases the pH in the outer part of layer. This influx of H^+^ causes the outer part of the layer to expand and soften. The reswelling of the outer layer is accompanied by the capacitive current, visible as a secondary wave in the current trace. As the upper part of the layer re-expands, the frequency decreases.

Before the potential is switched from negative to positive (right in Figure 6), the electrode surface is blocked, resulting in a local reduction in pH. A pocked with low pH and an expanded polymer is formed. The pocket decreases the coupling between the layer and the resonator surface, causing the frequency to increase. Subsequently, the barrier is completely removed. The entire layer experiences a uniformly low pH. At low pH, polyelectrolyte chains are more strongly charged, which leads to their expansion. The expansion lets the frequency decrease.

### 3.5 Interpretation in Terms of a Softness Parameter

Given the difficulties with explicit modeling, it would be desirable to have an inherently nongravimetric parameter that can be interpreted without the need for modeling. In the past, the ratio ΔΓ/(−Δ*f*) (the “Df ratio”,^66^ also: “acoustic ratio”^67^) has occasionally played that role.^68^ However, the Df ratio cannot be derived in this case because Δ*f* is not known in absolute terms after the resonator has been transferred between the two cells (as discussed in the first paragraph in Section 2.3).

**Figure 7:**
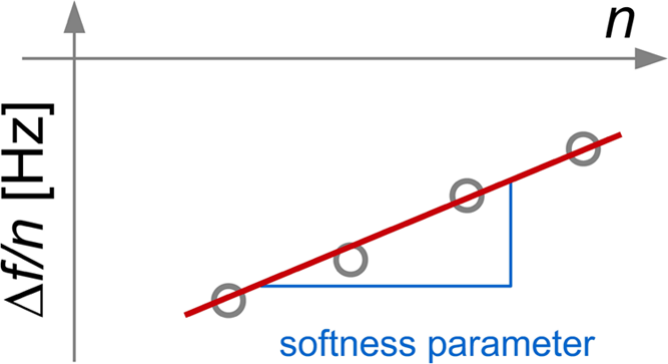
The softness parameter is defined as the slope of a plot of Δ*f*/*n* vs *n*. Changes in the softness parameter are independent of the reference state. An increasing slope indicates increasing softness.

We propose to use the spread in the values of Δ*f*/*n* between overtones instead. More precisely, we plot Δ*f*/*n* vs *n* for every single time and fit a straight line to these data (Figure 7). We call the slope of these lines “softness parameter”.

This softness parameter is model-free in the sense that no viscoelastic model is used in its derivation. However, the slope does have a concise meaning in the framework of the viscoelastic model. For thin and moderately soft layers deposited on a QCM sensor, the slope is proportional to the viscous compliance, *J*″ (Section 4.6.2 in ref (26)). The larger the slope, the softer the layer.

**Figure 8:**
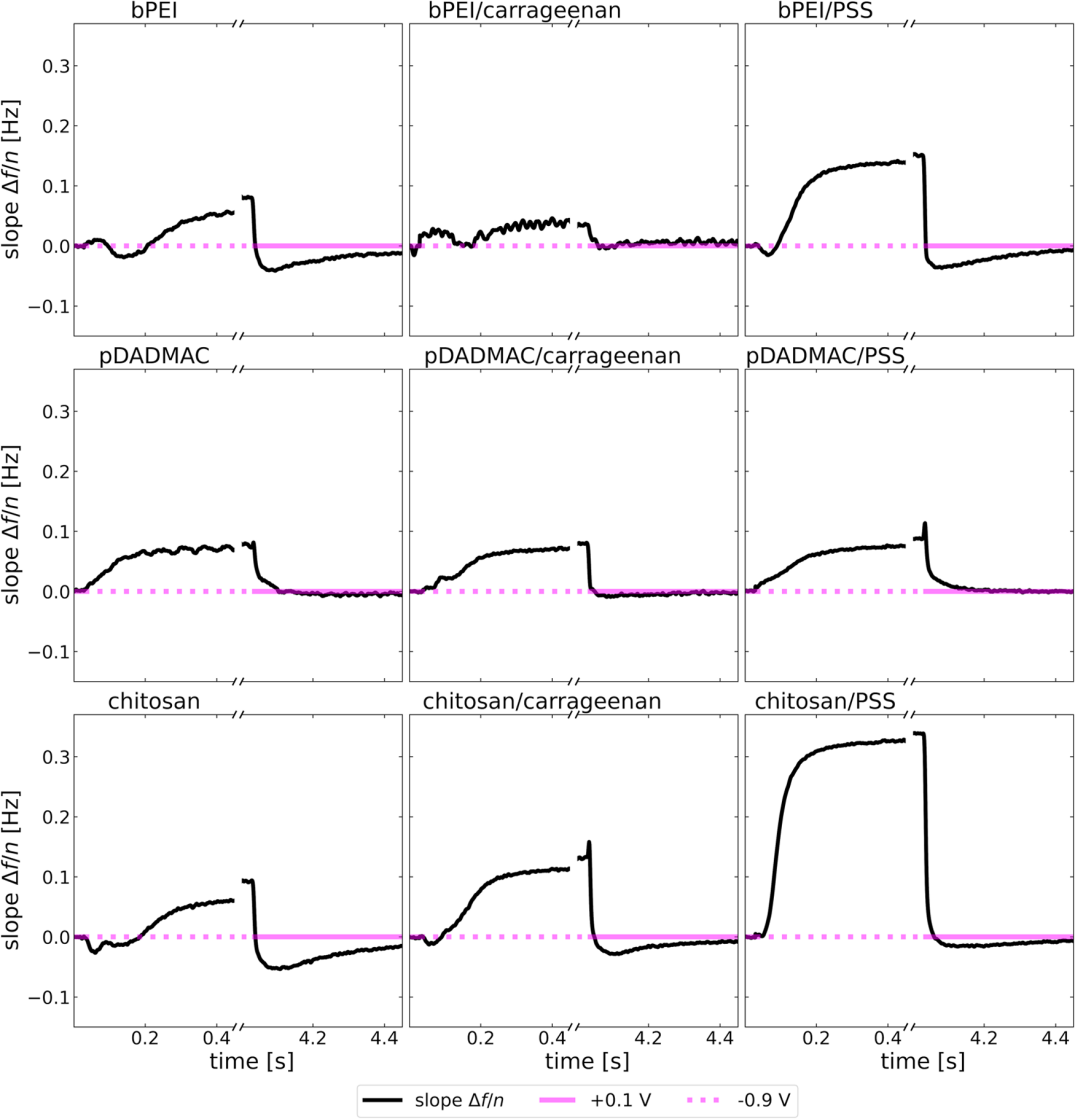
Slope in plot of Δ*f*/*n* versus *n*. Values of Δ*f*/*n* differ between overtones indicate nongravimetric behavior. The reference state (with no slope, by definition) is the state before switching to a negative electrode potential. By using this state as the reference state, it is assumed to be compact. Positive slopes correspond to a softer layer.

The variation of softness parameters in response to voltage modulation can be directly inferred from Figure 4. The different colors (corresponding to the different overtones) only spread out in the second (slower) process, and they only spread out, when the potential is negative. The softness parameter makes this observation quantitative. Note that the absolute values of the softness parameter again depend on the reference state. In the reference state, the softness parameter is zero by definition. However, *changes* in the softness parameter are independent of the reference state. The predominantly positive values of the slope in Figure 8 justify using the state before switching the voltage to negative as the pseudoreference state, representing the more compact state. That this state is not perfectly compact can be inferred from the slightly negative values of the slope sometimes seen after switching to a positive potential.

The softness parameter provides information which goes beyond Δ*f*/*n* and ΔΓ/*n*. The softness parameter hardly shows the initial contraction observed in Figure 4. The changes of the softness parameter do depend on the direction of the voltage step. Why, exactly, the layers become soft at negative potential—but not when the layers swell after the potential has become positive—is not entirely clear.

### 3.6 Streaming Potential Measurements of Adsorbed Layers

Streaming potential measurements allow to determine the changes in the ζ-potential of the surface caused by the sequential adsorption of anionic and cationic polyelectrolytes.

**Figure 9:**
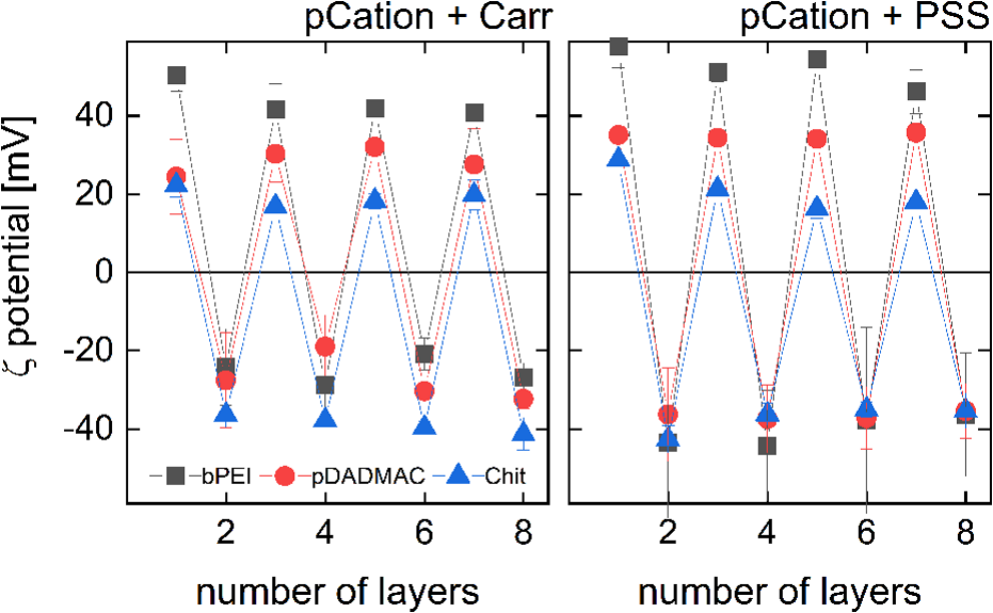
Dependences of the ζ-potential on the number of adsorbed layers. The streaming potential is found to be more negative for PSS than for Carr. Additionally, it depends less on the nature of the underlying polycation layer. This can be explained with interpenetration in the case of Carr, and the lack thereof in the case of PSS.

As observed previously,^62,69−71^ the consecutive adsorption of polycation and polyanion layers leads to a periodic reversal in the ζ-potential (Figure 9). However, there are systematic differences between the different materials. Here, we focus on interpreting one particular aspect, which corroborates the interpretation of the electroresponsivity. When PSS is the top layer, the ζ-potential of the substrate is always slightly more negative than in the case of Carr. This observation was made earlier by Elizarova and Luckham, who determined the ζ-potential of the multilayered capsules based on either pDADMAC/PSS or pDADMAC/Carr layers.^72^ This behavior is explained by intermixing of Carr with the underlying layers, which reduces the negative charge exposed to the bulk. Carr interpenetrates the underlying layers more than PSS because Carr is a hydrocolloid, while PSS is not. Carr chains tend to adopt an expanded conformation, while PSS forms a more compact layer. Due to the presence of the phenyl group and the high density of negatively charged sulfate groups in the side chains, PSS has a rigid structure. The use of PSS in multilayer preparation leads to the formation of a polyelectrolyte multilayer with a rough surface.^73^ Furthermore, the compactness of multilayers containing PSS, prepared under low ionic strength conditions, is evident from a monotonous decrease in water content as the number of layers increases from 2 to 8 in pDADMAC/PSS systems.^74^

Adsorption of the polycations bPEI, Chit, and pDADMAC changed the sign of ζ-potential of the substrate in all cases. Moreover, the effects on the ζ-potential follow the order bPEI > pDADMAC > Chit. This order can be explained as follows: the bulk ζ-potential of bPEI under the applied experimental conditions is higher than that of pDADMAC.^75,76^ Furthermore, the high polydispersity of bPEI^77^ leads to greater surface coverage. On the other hand, pDADMAC molecules, assuming an extended shape, adsorb mainly in a flat conformation, forming a compact, dense, and nonpermeable monolayer, which also exhibits a high ζ-potential.^76,78^ Finally, the Chit layer has a heterogeneous structure with uncovered regions, resulting in a significantly lower ζ-potential compared to pDADMAC.^62^ Interestingly, the ζ-potential of the subsequent Chit layers was higher than that observed for Chit deposited on silica.^79^

### 3.7 Contact Angles

**Figure 10:**
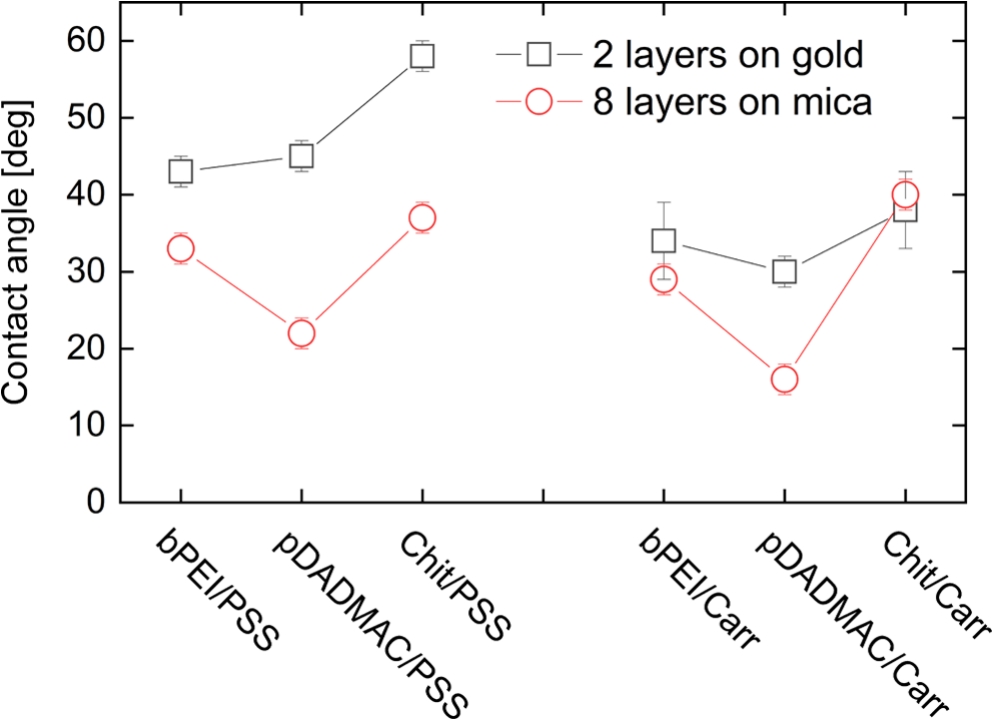
Water wets the substrates better when the top layer is Carr than when the top layer is PSS. Wettability improves further when the bottom layer is pDADMAC.

Figure 10 shows contact angles obtained with different top layers. When PSS terminated the multilayers, the contact angles increased from zero to 33, 22, and 37°, depending on the type of polycation adsorbed in the previous step. When Carr terminated the multilayers, the contact angles increased to 29°, 16° and 40° for bPEI, pDADMAC, and Chit as the anchor layer, respectively. The adsorption of bPEI/PSS, pDADMAC/PSS and Chit/PSS bilayers on gold resulted in a decrease in the contact angles to 43, 45, 58°, respectively. For Carr-terminated bilayers, the values were 34, 30, and 38°.

With one exception, the contact angles obtained when the top layer was Carr were lower than those obtained with a top layer of PSS. This finding presumably is related to the hydrophobicity of the phenyl ring in PSS. Most striking is the fact that water wets the surfaces best when the bottom layer is pDADMAC. As discussed in Section 3.6, pDADMAC – not being a hydrocolloid–adsorbs in a compact form and therefore does not allow for interpenetration by layers adsorbed on top of it. Interpenetration reduces the net negative charge exposed at the film–air interface, thereby reducing wettability. The contact angles for pDADMAC/PSS systems are consistent with those obtained by the Warszynski group.^80^ These authors also found that films terminated by a polyanion are more hydrophilic than those terminated by a polycation.

Furthermore, the contact angles measured for Chit/Carr pairs agree well with the data obtained by Pinheiro et al. for multilayers deposited on polyethylene terephthalate.^81^ This suggests that surface wettability is independent of the substrate type. It is also worth highlighting that in our studies, the effect is very pronounced even when the layers were obtained from polyelectrolyte concentrations as low as 5 mg L^–1^. Carr-terminated films may be useful for polymer-based surgical implants, for which one of the obstacles to effective use is their hydrophobic nature.^82,83^

## 4 Discussion

### 4.1 Methodology, Comparison with Established Characterization Methods

As shown above, the interpretation of the QCM data becomes more conclusive when electroresponsivity is exploited. Additionally, changes in the softness parameter (Figures 7 and 8) are easier to interpret than the shifts in frequency and bandwidth themselves (Figure 4). Interpretation is further aided by measurements of the streaming potential and the contact angles.

Studying electroresponsivity with an EQCM is not particularly difficult per se. It has also been carried out on ionic liquids.^84,85^ However, the interpretation can be challenging, which is among the drawbacks. Both physical effects (such as recharging of the double layer^86^) and genuinely electrochemical effects (redox processes at the electrode^87^) contribute. If the redox processes involve water, the local pH changes,^88^ which affects the conformation of weak polyelectrolytes.^89^ The analysis is further complicated by the possibility of vertical structure in the layer,^90^ such as a thin, compact layer at the bottom. The QCM is largely insensitive to structure with characteristic scales much smaller than the penetration depth of the shear wave. Another complication is viscoelastic dispersion, that is, the dependence of viscosity on frequency. The comparison between overtones (which have different penetration depths of the shear wave) might, in principle, provide insight into the structure, but distinguishing effects of structure from effects of viscoelastic dispersion is difficult. Put differently, viscoelastic dispersion is interesting because it provides access to relaxations in the medium, but this analysis becomes problematic when effects of relaxation are mixed with effects of structure.

In this context, it is helpful to run the EQCM as fast as possible because kinetics provides an additional source of information. This concept is familiar from dynamic electrochemistry.^91^ In particular, double layer recharging in small-molecule electrolytes is known to occur within the “RC-time”, where *R* is the bulk resistance and *C* is the double layer capacitance.^91^ In other experiments, a QCM response has occurred on that same time scale.^48^ When a QCM-D responds to a voltage step within the RC-time, the RC-time is only discernible if it is longer than the intrinsic response time of the quartz resonator (which is (2πΓ)^−1^ ≈ 200 μs).^92^ If the concentration is too high (higher than about 100 mM), the RC-time is not resolved.

Unrelated to kinetics, accumulation and averaging over many modulation cycles also help. Accumulation and averaging much improve the precision. Improved precision makes it easier to determine the kinetics. Also, it is of importance when comparing plateau values between samples.

As always, combination with other techniques may help. Measurements of the streaming potential and the contact angles were employed here. Electrochemical impedance spectroscopy (EIS) comes for free in the context of the EQCM.^93^ Monitoring the current as in chronoamperometry,^91^ (as done here), is related to a time-domain version of EIS. Optical reflectometry can be done with moderate effort.^94,95^ However, optical reflectometry suffers from the same drawbacks as acoustic reflectometry (that is, as the QCM, see the discussion in Section 1.3). The ultimate technique for investigating small-scale structure is neutron reflectometry.^96^ However, neutron reflectometry is expensive and has much lower time resolution than the EQCM. Various other methods of operando characterization in electrochemistry deserve a mention.^97^ Neither X-ray spectroscopy^98^ nor Raman spectroscopy^99^ are particularly useful here, as changes in conformation of polymer chains leave few traces in these data sets.

### 4.2 Mechanisms of Electroresponsivity

The electroresponsivity observed when modulating the electrode potential is too slow to be explained by charge inversion in the diffuse double layer (as seen in reference experiments on bare gold surfaces^48^). Given that the dynamics of polymers is slower than those of small molecules,^100^ the response observed in these experiments should be attributed to changes in the conformation of the adsorbed chains. Chit and bPEI monolayers exhibit a two-stage response: the faster process resembles a contraction, while the slower process resembles an expansion. Notably, the sign of these effects is independent of the sign of the voltage step. The explanation of this complicated behavior (Section 3.4) is based on a collapsed layer acting as a barrier against the transport of H^+^.^101^ Only pDADMAC (not a hydrocolloid) shows a one-step response, the sign of which changes with the direction of the voltage step. While we are not aware of explicit mention of such a barrier in the literature, the experiments described in ref (4). can be interpreted using this framework. The authors of that study also observed an asymmetry between the kinetics of swelling and deswelling, albeit on a longer time scale.

The strongest electroresponsivity by far is seen for Chit covered with PSS, followed by bPEI covered with PSS. In contrast, pDADMAC exhibits only minor effects, because it is a strong polyelectrolyte whose charge is pH-independent. The greater electroresponsivity associated with a top layer of PSS, as opposed to Carr, is attributed to differences in interpenetration.^102^ Interpenetrated polyelectrolytes of opposite charge form strong electrostatic links between oppositely charged groups,^103^ which works against swelling and deswelling. The lack of interpenetration in the case of PSS is also evidenced by the contact angles and the streaming potential.

Interestingly, coating layers of bPEI or Chit with PSS does not only leave electroresponsivity intact but actually enhances it. The enhancement is far from obvious. A possible explanation is that PSS, being compact and hydrophobic, forms a barrier against the transport of H^+^. Pictorially speaking, PSS forms a “lid” on top of Chit or bPEI, which delays equilibration between the near-surface pH and the bulk pH. Consequently, the pH inside the first layer is changed more significantly in the presence of the cover layer, which increases the electroresponsivity. We are not aware of a previous mention of such an effect.

### 4.3 Implications for Materials Design

Hydrocolloids are materials of significant practical importance.^29^ They are often used as additives or coatings,^27^ valued for their biocompatibility and open structure, which is caused by the tendency to form physical cross-links. Some hydrocolloids, including Chit and Carr, are weak polyelectrolytes, while others are not. As was demonstrated in this study, thin layers of Chit and Carr exhibit both pH-responsiveness and electroresponsiveness. However, both effects became weaker when layers of opposite charge were deposited one on top of the other and when these layers interdigitate. This set of phenomena is well-documented in polyelectrolyte complexes (mixtures of strong or weak polyelectrolytes of opposite charge).^104^ They deserve renewed attention in the context of multilayer coatings^14^ and their functions.^105^

## 5 Conclusions and Outlook

While the structure and the dynamics of adsorbed hydrocolloids are undoubtedly complex, they can be elucidated using the combination of techniques mentioned above. Electroresponsivity is primarily driven by pH. There is an asymmetry in the kinetics of expansion and contraction, which is caused by the collapsed layers forming a barrier. Furthermore, electroresponsivity is reduced by interpenetration between oppositely charged layers.

An obvious expansion of the work is to carry out adsorption under controlled electrical potential. That statement includes layer-by-layer deposition. The degree of openness can be tuned electrically. One might even produce laterally structured layers by using different electrodes at different potentials. Lastly, this work has practical implications for the use of hydrocolloids as food coatings. The degree of interpenetration between different layers depends on the pH, at which they are formed. Our results confirmed that electroresponsivity, combined with streaming potential and contact angle measurements, can be a useful tool for determining the mechanical and the physicochemical properties of polyelectrolyte multilayers, which can form either rigid or gel-like scaffolds. We believe that this insight holds significance for designing novel intelligent polyelectrolyte-based dressings that, for example, may accelerate the healing of acute and chronic wounds.

## Supporting Information Available

Composition of carrageenan; fits to the adsorption kinetics of bPEI, starting from two different guess values, close to the two minima on the upper left in Figure 3 in the main text; and electric current measurements presented alongside the QCM response (PDF)
