# Supplementary material for: Swelling Degree of Polyelectrolyte Layers Determined by an Electrochemical Quartz Crystal Microbalance
Source: Biomacromolecules. 2025 Jan 22;26(2):914–28. doi: 10.1021/acs.biomac.4c01205 (PMC11815823; doi:10.1021/acs.biomac.4c01205)
Supplement: Supplementary file 1 — bm4c01205_si_001.pdf [file bm4c01205_si_001.pdf]

Swelling Degree of Polyelectrolyte Layers Determined by an Electrochemical Quartz Crystal Microbalance

Christian Leppin[1], Agata Pomorska[2*], Maria Morga[2], Pawel Pomastowski[3,4], Piotr Fijałkowski[3], Aneta Michna[2], Diethelm Johannsmann[1*]

[1]Institute of Physical Chemistry, Clausthal University of Technology, Arnold-Sommerfeld-Str. 4, 38678 Clausthal-Zellerfeld, Germany.

[2]Jerzy Haber Institute of Catalysis and Surface Chemistry, Polish Academy of Sciences, Niezapominajek 8, PL-30239 Krakow, Poland.

[3]Centre for Modern Interdisciplinary Technologies, Nicolaus Copernicus University, Wilenska 4, 87-100 Toruń, Poland.

[4]Department of Inorganic and Coordination Chemistry, Nicolaus Copernicus University in Toruń, Gagarina 7, 87-100 Toruń, Poland.

*Correspondence: agata.pomorska@ikifp.edu.pl, johannsmann@pc.tu-clausthal.de

SUPPORTING INFORMATION

1. Composition of carrageenan

1.1 Matrix-assisted laser desorption/ionization time-of-flight mass spectrometry (MALDI-TOF/TOF) and dynamic light scattering (DLS) analysis of carrageenan (Carr)

For the MALDI-TOF/TOF analysis, Carr was dissolved in cold water (Milli-Q Reference, Merck) at a concentration of 1 mg/mL and subjected to ultrasonication using an ultrasonic cleaner (Polsonic, Poland). Additionally, a second sample was prepared similarly to the first, but after dissolution and ultrasonication, this sample underwent ultrafiltration (UF). UF was performed using Amicon-type tubes (Merck) with a 3,000 Da cut-off membrane. The dissolved sample was transferred to a 4 ml Amicon tube and centrifuged for 15 minutes at 4,000 rpm using a Centrifuge 5810 R (Eppendorf, Germany). Subsequently, the sample was washed by adding 2 mL of distilled water, followed by centrifugation. The washing step was repeated three times, with the final centrifugation lasting for 30 minutes. The prepared samples (before and after UF) were used for further analysis. The permeate obtained from UF was also collected and retained for analysis.

Carr samples were mixed in equal volumes with a matrix. A saturated solution of α-cyano-4-hydroxycinnamic acid (HCCA) in TA30 (30:70 v/v ACN:0.1% TFA in water) was used as the matrix. After mixing the sample with the matrix, the resulting solution was spotted in a volume of 1µL onto a MALDI target plate MTP 384 (Bruker Daltonics, Germany). MALDI-TOF MS analysis was performed using an ultrafleXtreme II instrument (Bruker Daltonics, Germany) equipped with a modified neodymium-doped yttrium aluminum garnet (Nd:YAG) laser operating at 355 nm with a frequency of 2 kHz. Measurements were conducted in positive ion mode. The first accelerating voltage was set at 25.09 kV, and the second ion source voltage at 23.42 kV. The spectra were collected manually, with 500 shots in 10 repetitions in a buffer, and three repetitions overall. The measurement range spanned from 1 to 2000 m/z in reflectron positive mode, and from 2000 to 20,000 m/z, as well as 20,000 to 200,000 m/z in linear positive mode. For calibration of higher m/z values, Protein

Standard II (Bruker Daltonics, Germany) was used, while cesium iodide (CsI) (Sigma Aldrich, Germany) was employed for the lower mass range (m/z < 2 000).

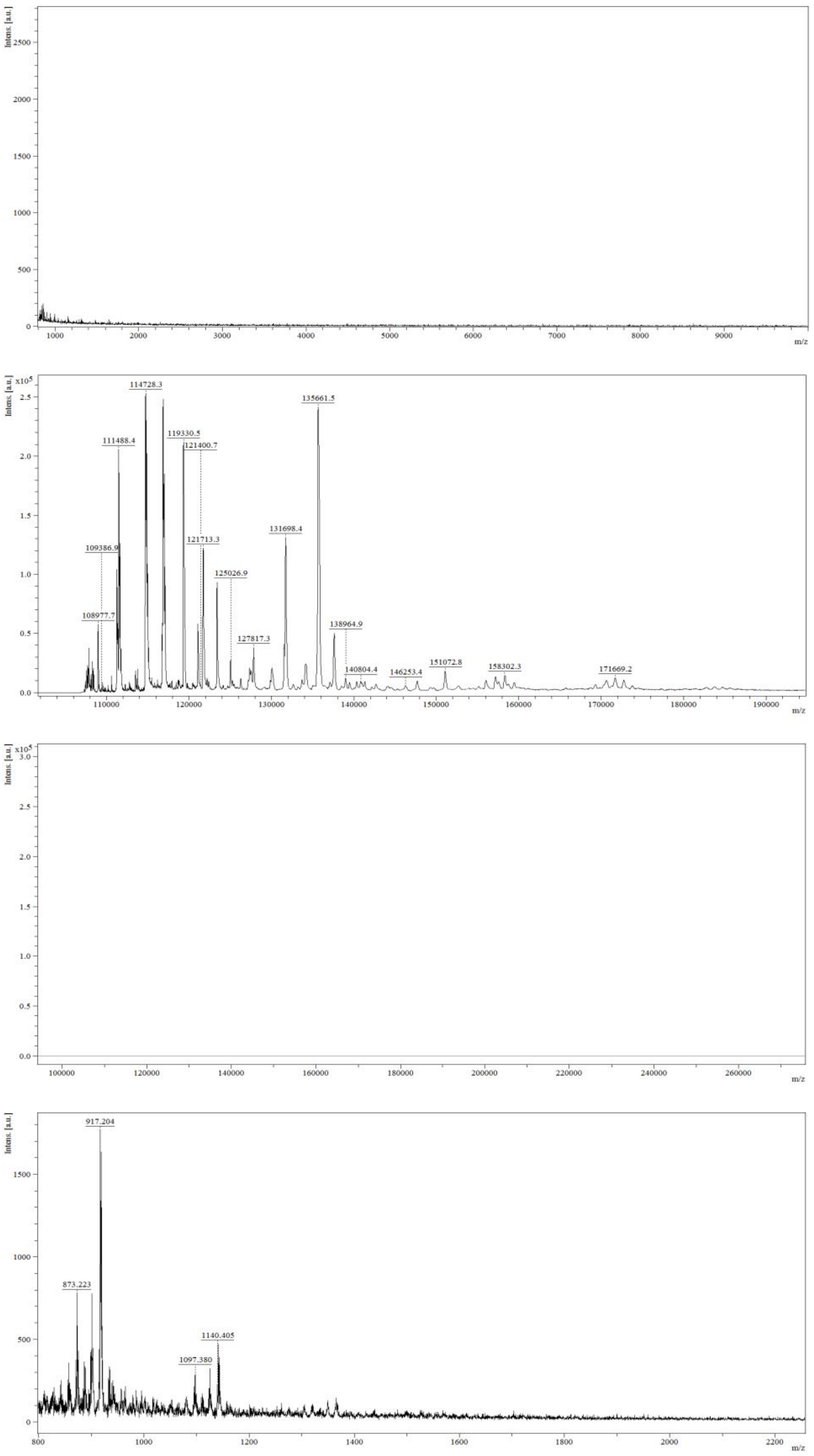

Fig. S 1: Results from mass spectroscopy analysis of Carr. The figures, presented in order from top to bottom, correspond to the following: before UF; after UF; before UF; and permeate.

For Carr samples (before UF, after UF, and permeate), hydrodynamic size and zeta potential measurements were carried out using dynamic light scattering (DLS) by Zetasizer Nano Series

instrument (Malvern, United Kingdom). Prior to measurements, the samples were subjected to ultrasonication for approximately 1 minute at 25°C. Hydrodynamic size and zeta potential measurements were conducted three times, each consisting of three repetitions, at a constant temperature of 25°C.

MALDI-TOF/TOF MS analysis for the sample before UF did not yield significant peaks at higher m/z values. For lower m/z values, a noticeable baseline drift was observed within the m/z range of 2 – 12 kDa. This drift is likely attributed to suppression effects caused by chloride salts and low molecular weight compounds. The presence of chloride salts is further confirmed by the reaction with silver nitrate, which produced a white precipitate of AgCl. For the sample subjected to the UF process, mass peaks were obtained in the range of m/z 108 – 171 kDa. In contrast, the permeate sample displayed three peaks at m/z values of 873.22, 917.20, and 1097.38. The structure of Carr consists of a repeating disaccharide unit composed of a 3-linked β-D-galactopyranosyl residue and a 4-linked 3,6-anhydro-α-D-galactopyranosyl residue.[1,2] Individual Carr structures, of which at least 15 are known, differ from each other in the presence of a 3,6-anhydro bridge and the position of sulfate substituents.[1] In the mass spectrum obtained for the Carr sample after the UF process, mass peaks in the range of m/z 108-17 kDa were observed. These peaks arise from polysaccharide fragmentation. Differences between them, in individual fragments, may provide insights into the structure of the Carr being studied. Notably, within the m/z range of 108 977.662 – 114 728.292, cyclically repeating peaks with a constant mass difference of approximately 2,875 Da were identified. This mass difference may correspond to the fragment [(A-G4SNa-A)5 + Na]+, calculated based on the work of Aguilan et al.[1] This fragment is consistent with the structure of κ-carrageenan.

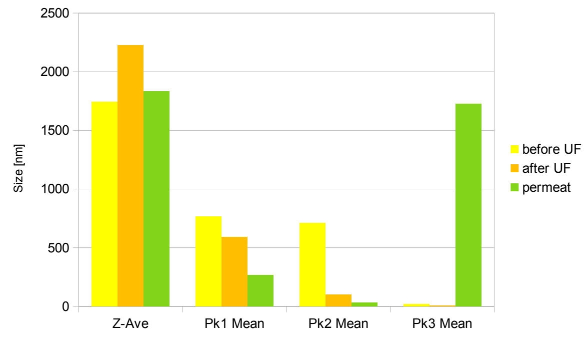

Fig. S 2:
The hydrodynamic size of Carr determined by DLS techniques.

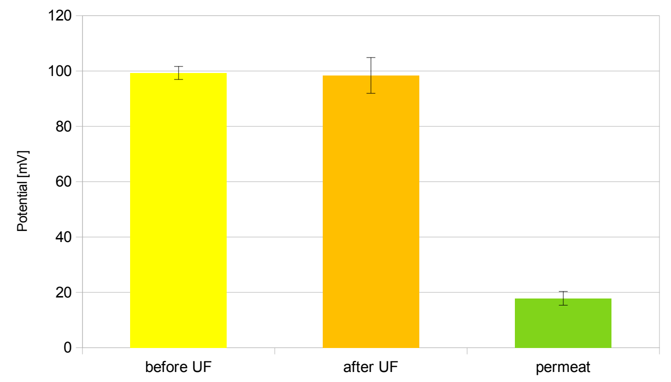

Fig. S 3:
Zeta potential of Carr samples. Detailed bulk characterization of Carr allows us to determine the exact composition of this polyanion. It was found that the commercially obtained polyanion is a mixture of κ and ι-carrageenans.

The results of hydrodynamic size measurements are presented in Fig. S 2. The average Z-average value obtained from nine measurements for samples before UF, after UF, and permeate were 1745, 2225, and 1833 nm, respectively. The values of individual peaks (Pk) are also provided.

For the sample before UF, the average values were as follows:
Pk1: 765.6 nm (74.2% area)
Pk2: 710.5 nm (22.4% area)

Pk3: 20.19 nm (3.4% area)
For the sample after UF, the parameters for individual peaks were:
Pk1: 590.9 nm (75.4% area)
Pk2: 99.65 nm (22.3% area)
Pk3: 8.131 nm (2.0% area)

For the permeate, the peak values were:
Pk1: 264.5 nm (86.0% area)
Pk2: 32.88 nm (9.7% area)
Pk3: 1728 nm (4.3% area)

The DLS measurement results indicate a reduction in the hydrodynamic radius of Carr for Pk1, Pk2, and Pk3 for the UF-treated sample. This decrease may be attributed to the removal of ions during the ultrafiltration process.

The results for zeta potential are presented in Fig. S 3. The zeta potential values for samples before and after UF are consistent and amount to −99.3 and −98.4 mV, respectively (absolute values are shown in the figure). For the permeate, the zeta potential value was significantly lower at −17.83 mV. A high absolute value of the zeta potential indicates the high stability of the Carr solution. Solutions with an absolute zeta potential value exceeding 30 mV are considered stable, and values above 60 mV indicate high stability.

2. Fits to adsorption kinetics of bPEI, starting from two different guess values, close to the two minima on the upper left in Fig. 3 in the main text

Fig. 3 in the main text shows broad χ^2 landscapes. The χ^2 landscapes for bPEI, in particular, exhibit two minima. The solution found by the fitting process, depends on the starting value. This argument is further supported by Fig. S 4, which shows the thickness and the viscoelastic constants versus time, based on $\Delta f/n$ and $\Delta\Gamma/n$ as determined during the adsorption of bPEI. Depending on whether the initial guess value for the film thickness corresponds to the left of the right minimum in Fig. 3 of the main text, different solutions are found. This ambiguity exists for the entire adsorption kinetics.

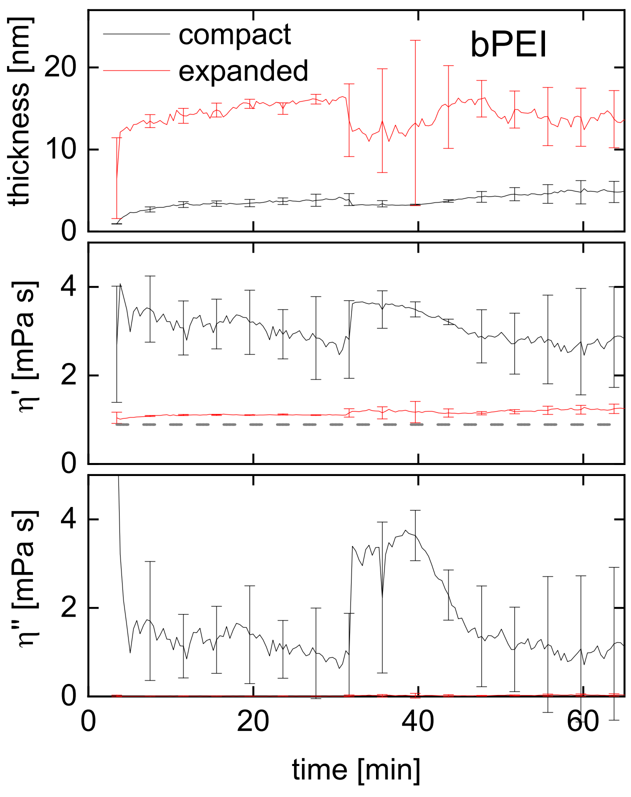

Fig. S 4:
Fits of the viscoelastic model to the adsorption experiment shown in the upper left of Fig. 2 in the main text. The entire adsorption curve changes depending on the thickness used as the guess value (compact or expanded). Values of β' and β'' are not shown (but were β' and β'' were also free parameters).

3. Electric current shown together with the QCM response

Fig. 5 in the main text shows the current response together with the QCM response for the case of

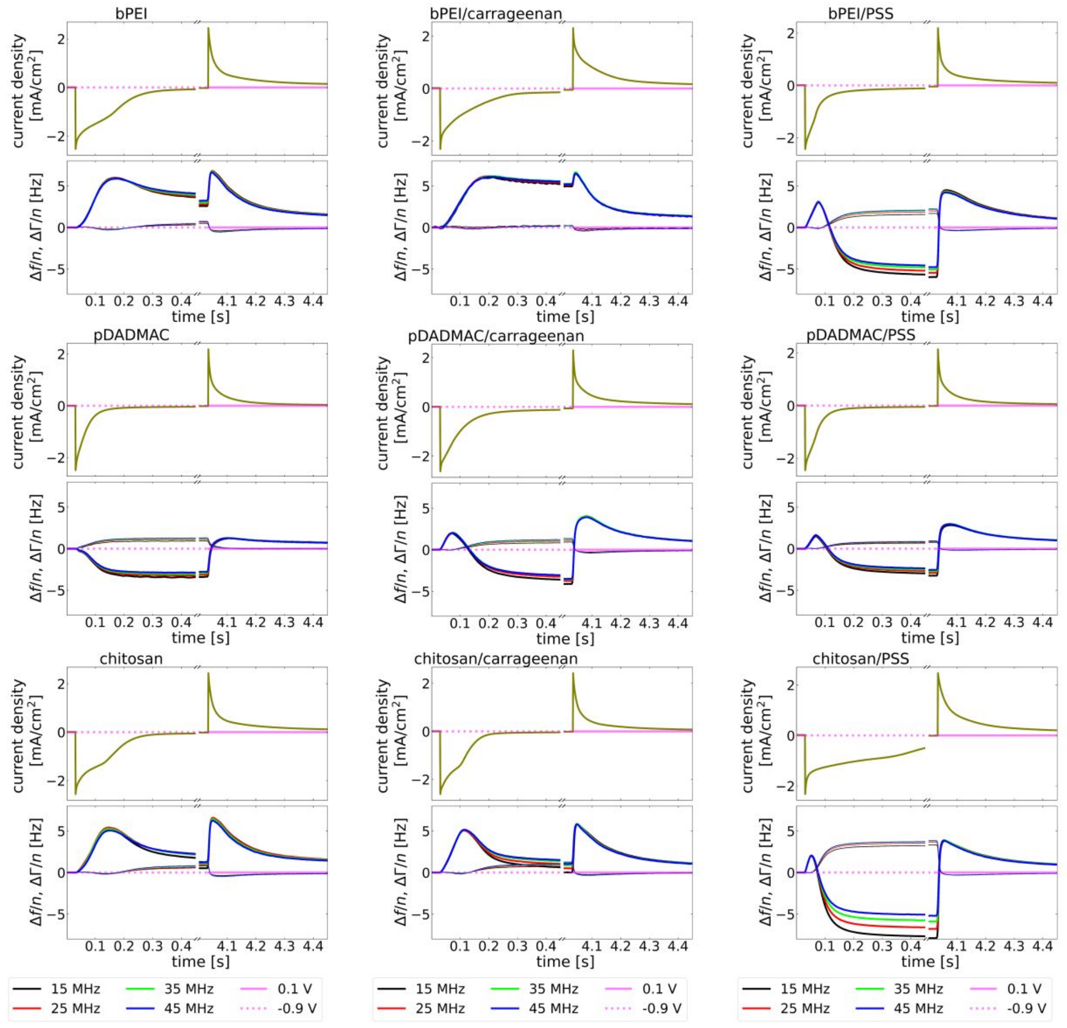

Fig. S 5:
Current densities together with the QCM responses for the entire data set from Fig. 4 in the main text.

bPEI. Fig. S 5 reports analogous graphs for data from Fig. 4 in the main text.

REFERENCES

[1] Aguilan, J. T.; Dayrit, F. M.; Zhang, J.; Niñonuevo, M. R.; Lebrilla, C. B. Structural Analysis of κ-Carrageenan Sulfated Oligosaccharides by Positive Mode Nano-ESI-FTICR-MS and MS/MS by SORI-CID. *J. Am. Soc. Mass Spectrom.* **2006**, *17* (1), 96–103. https://doi.org/10.1016/j.jasms.2005.09.009.

[2] Aguilan, J. T.; Broom, J. E.; Hemmingson, J. A.; Dayrit, F. M.; Montaño, M. N. E.; Dancel, M. C. A.; Niñonuevo, M. R.; Furneaux, R. H. Structural Analysis of Carrageenan from Farmed Varieties of Philippine Seaweed. **2003**, *46* (2), 179–192. https://doi.org/10.1515/BOT.2003.018.
